# Brain Computer Interface on Track to Home

**DOI:** 10.1155/2015/623896

**Published:** 2015-06-08

**Authors:** Felip Miralles, Eloisa Vargiu, Stefan Dauwalder, Marc Solà, Gernot Müller-Putz, Selina C. Wriessnegger, Andreas Pinegger, Andrea Kübler, Sebastian Halder, Ivo Käthner, Suzanne Martin, Jean Daly, Elaine Armstrong, Christoph Guger, Christoph Hintermüller, Hannah Lowish

**Affiliations:** ^1^Barcelona Digital Technology Center, C/Roc Boronat 117, 08018 Barcelona, Spain; ^2^Graz University of Technology, Inffeldgasse 13/4. OG, 8010 Graz, Austria; ^3^Institute of Psychology, University of Würzburg, Marcusstraße 9-11, 97070 Würzburg, Germany; ^4^Faculty of life and Health Sciences, University of Ulster, Jordanstown BT370QB, UK; ^5^Cedar Foundation, 31 Ulsterville Avenue, Belfast BT9 7AS, UK; ^6^g.tec Guger Technologies OG, Sierningstrasse 14, 4521 Schiedlberg, Austria; ^7^Telehealth Solutions, Building 6, Suite 7a, Croxley Green Business Park, Hatters Lane, Watford WD18 8YH, UK

## Abstract

The novel BackHome system offers individuals with disabilities a range of useful services available via brain-computer interfaces (BCIs), to help restore their independence. This is the time such technology is ready to be deployed in the real world, that is, at the target end users' home. This has been achieved by the development of practical electrodes, easy to use software, and delivering telemonitoring and home support capabilities which have been conceived, implemented, and tested within a user-centred design approach. The final BackHome system is the result of a 3-year long process involving extensive user engagement to maximize effectiveness, reliability, robustness, and ease of use of a home based BCI system. The system is comprised of ergonomic and hassle-free BCI equipment; one-click software services for Smart Home control, cognitive stimulation, and web browsing; and remote telemonitoring and home support tools to enable independent home use for nonexpert caregivers and users. BackHome aims to successfully bring BCIs to the home of people with limited mobility to restore their independence and ultimately improve their quality of life.

## 1. Introduction

Research efforts have improved brain computer interface (BCI) technology in many ways and numerous applications have been prototyped. Motivated by the aim of restoring independence to individuals with severe disabilities, the focus has centred on developing applications [1–3] for communication [4, 5], movement control [6, 7], environmental control [8], locomotion [9], and neurorehabilitation [10–12]. Until recently, these BCI systems have been researched almost exclusively in laboratories. Home usage has been demonstrated, though only with on-going expert supervision. A significant advance on BCI research and its implementation as a feasible assistive technology (AT) is therefore the migration of BCIs into people's homes to provide new options for communication and control that increase independence and reduce social exclusion.

In this context, the EU project BackHome (http://www.backhome-fp7.eu/) is aimed at moving BCIs from being laboratory devices for able-bodies users toward practical devices used at home by people with severe limited mobility. This requires a system that is easy to set up, portable, and intuitive. Thus, BackHome aims to develop BCI systems into practical multimodal ATs to provide useful solutions for communication, web access, leisure, cognitive stimulation, and environmental control and to provide this technology for home usage with minimal support.

The project was designed with a user centred design (UCD) approach at the heart of the whole R&D process in order to move BCIs from the lab towards systems that are easy to use and can be operated by nonexpert caregivers and target end users at home. In this R&D field, UCD was proposed to bridge the translational gap between BCI systems and their target end users [13]. In BackHome, UCD has been used for the first time throughout all development stages of a multifunctional BCI and here lies the main innovation driver of the project. Driven by this innovative UCD approach towards independent home use, BackHome has achieved five key innovations presented in this paper advancing the current state of the art: (i) a modular and distributed architecture able to meet the requirements of a multifunctional BCI with remote home support; (ii) a novel BCI equipment with practical electrodes aimed at setting a new standard of lightness, autonomy, comfort, and reliability; (iii) easy to use software tailored to people's needs to manage a complete range of multifunctional applications finely tuned for one-click command and adaptive usage; (iv) a telemonitoring and home support system to remotely monitor and assist BCI independent use; and (v) a web-based application for therapists which offers remote services to plan and monitor BCI-based cognitive rehabilitation and pervasively assess the use of the system and the quality of life of the individual.

This paper presents the UCD process employed to research and develop a multifunctional BCI system, including the collection of user requirements, the iterative development process together with the analysis of test results, and feedback recommendation from able-bodied and disabled end users. Last but not least, the paper presents the outcome of this UCD process, which is the final BackHome system that is currently installed in target end users' homes for final testing and evaluation.

The rest of paper is organized as follows.  Section 2 summaries the state of the art of BCI systems with particular references to those that have been deployed at users' home. In Section 3, we outline the BackHome project objectives and introduce the final system describing the overall architecture and services.  Section 4 is the core section of the paper, depicting the materials and methods, an overview of the newly adopted UCD methodology to this field, ethical considerations, user requirements collected in the first stage of the project, and the evaluation of prototypes by the various project stakeholders, with a discussion of specific recommendations for implementation matched to user requirements.  Section 5 presents the final system, the design, and implementation details for the five main innovative elements of the system, that is, architecture and practical electrodes; easy to use software; telemonitoring and home support and therapist station; and first results obtained with both able-bodied and disabled end users.  Section 6 concludes by discussing the impact of the results achieved so far and describes the next steps, when we aim to assess the impact of the BackHome system with target end users in their own homes, future work, and technology transfer to the market.

## 2. Literature Review

Until recently, BCI research focused primarily on validating devices with healthy volunteers in laboratory settings. This has begun to change [14–17]. These efforts have shown that BCIs can provide solutions for people in their homes [4, 18]. However, efforts to provide BCI solutions for home usage have highlighted one major problem: dependence on outside support. Thus, the key problems issued with BCI systems at home—and also the two principal reasons why home users need support—are difficult electrode set-up and need of software support [19]. In [20] it is shown that users do indeed identify electrodes as the top problem for home support and that improved software that integrates varied functionalities without too much problems is also essential [21].

In the following subsections, we briefly summarize the main work on practical electrodes, easy to use software, and telemonitoring and home support for independent home use of BCIs.

### 2.1. Practical Electrodes

To advance existing BCI systems into a more practical solution for home use it is necessary to improve the hardware and software of existing BCI systems. Since the majority of BCIs are based on electroencephalography (EEG) it is one of the main objectives to improve the sensors, namely, the EEG electrodes, which capture brain activity. One of the biggest problems with most noninvasive BCI systems is the need for sensors that rely on electrode gel. Surveys of able-bodied and disabled users [20, 22] reveal that users dislike conventional gel-based systems and their associated preparation time and inconvenience. Several different types of electrode systems are available on the market, and additionally many research groups tried to improve specific aspects to improve the applicability of the electrodes [23–25] or wireless signal transmission. There is room for improvement of user acceptance of BCI equipment by advancing ergonomics of caps, using reliable dry electrodes, wireless light amplifiers, and enable short and easy setup. All these features have been taken into account along the project.

### 2.2. BCI Software

Software currently adopted for research on BCI offers the researchers many options and functionalities [26]. Nevertheless, they are very hard to be used from nonexperts people. Thus, UCD principles have to be applied to optimize the system around how users can use the system in contrast to force the users to change their behavior to be able to use the system. The UCD is standardized in the ISO 9241-210 [27].

Three main aspects have to be considered to assess the usability of a BCI system: (i) the effectiveness, (ii) the efficiency, and (iii) the satisfaction of the user with the system. The effectiveness is indicated by the accuracy of the BCI system (correct selections of total selections). Accuracies lower than 70% are not acceptable for BCI systems [4]. The efficiency is mainly assessed with the information transfer rate (ITR) [28]. In addition to the accuracy, the ITR includes the information content within each selection (amount of possible selections) and the time needed per selection.

Different questionnaires are used to determine the satisfaction of the user with a system. For example, Zickler et al. introduced in [20] an extension of the QUEST 2.0 [29] for BCI usage. This is a powerful tool to get feedback from the user about her/his satisfaction with the BCI system. Another simple method is to let the users fill out a visual analogue scale (VAS) about satisfaction.

According to the UCD, the users' feedback is used to improve the current version of the software or to develop a new, better version. Consequently, using UCD principles make the BCI software easier to use and therefore more accepted by the target users.

### 2.3. Telemonitoring and Home Support

Home sensor technology may create a new opportunity to reduce healthcare costs by helping people to stay healthy longer as they age. An interest has therefore emerged in using home sensors for health promotion [30]. This interest has resulted in research and development of telemonitoring and home support systems (TMHSS), which are aimed at remotely monitoring patients who are not located in the same place as the health care provider. Those support systems allow patients to remain living in their own home [31]. Continuous monitoring and follow-up of patients are a convenient way for patients to avoid travelling to a health care institution and to perform some of the healthcare tasks by themselves, thus reducing the overall costs of healthcare [32, 33].

In summary, a TMHSS allows (i) improving the quality of clinical services, by facilitating the access to them, helping to break geographical barriers; (ii) keeping the objective of a patient-centred assistance, facilitating the communication between different clinical levels; (iii) extending the therapeutic processes beyond hospitals or primary care centres, like at patient's home; and (iv) saving unnecessary costs and achieving a better costs/benefits ratio.

In the literature, several TMHSSs have been proposed [34–36]. As targeted for BCI users, some work has been presented to provide Smart Home control [37–40]. Nevertheless, to our best knowledge, telemonitoring and home support has not been integrated yet with remote BCI systems as a way to allow remote communication between therapists and users and to improve remote support [18].

## 3. The BackHome Project

BackHome focuses on restoring independence of people that are affected by severe motor impairment due to acquired brain injury or disease, with the overall aim of increasing inclusion. In order to keep the user engaged, BackHome continuously provides feedback to therapist for the follow-up and for personalization and adaptation of rehabilitation plans.

The BackHome system integrates the developments undertaken through a UCD life cycle, main innovation driver of the project towards the goal of advancing a practical solution for independent home use. It includes a user station with wireless EEG biosignal acquisition system, a dynamic environmental sensor network, and an easy to use BCI software to access a range of services to facilitate activities of daily living. Additionally, the therapist station provides services for remote planning and assessment of user activity, rehabilitation tasks, and quality of life indicators.

### 3.1. System Architecture

**Figure 1:**
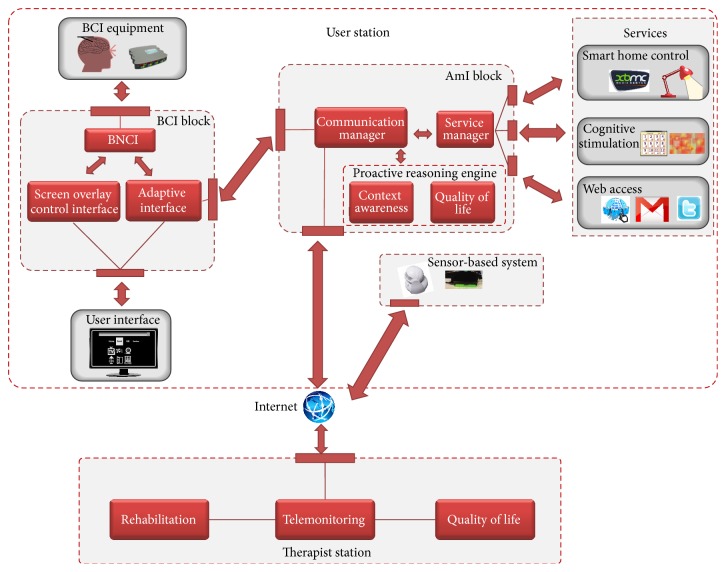
BackHome architecture overview.

Figure 1 shows the high level modular and distributed architecture of the BackHome system, accessed through two interconnected stations: (i) user station and (ii) therapist station.

The end user interacts with the user station, the BCI-based subsystem which comprises the modules responsible for the BCI components (a screen user interface together with a BCI block and BCI equipment) connected to the services of Smart Home control, cognitive stimulation, and web access through the AmI block, which holds the intelligence of the system. To execute and control its functionalities the user station, the BCI equipment, home sensors, and actuators must be installed and completely integrated at the user's home. The AmI block is the central control and intelligence component of the user station. It communicates and interacts with all other components of the system. It provides control to all services for Smart Home control, cognitive stimulation, and web access, receives the user selections from the BCI system, and reports indicators relevant to the user assessment to be checked out through the therapist station. The BCI block is connected to the user through the BCI hardware and the user interface, which displays the stimuli for the BCI and the interfaces of all the services. With the proposed BCI, all applications can be operated via control matrices that were first proposed in [41] for a spelling system. As control signals the BCI uses event-related potentials (ERPs) that can be extracted from the EEG. Of these ERPs the P300 is often the most prominent [42]. During stimulation, rows and columns of the matrix are highlighted in random order. To operate the system, users are asked to attend to the symbol (e.g., a letter or a command) in the matrix that they want to select and silently count whenever it is highlighted. The rows and columns including the target symbol elicit the ERP response. Thus, the system can identify the target symbol as the symbol at the intersection of the row and column that elicited the P300 response and execute the desired action. The BCI components together allow the user to control the user station with services and actuators and receive feedback from sensors and services.

The therapist station is a web-based easy to use service which allows a remote therapist, teleassistance service operator, or another professional to access information stored in the cloud and gathered from users and sensors around them: users' inputs, activities, selections, and sensor data. This information takes the form of system usage reports, rehabilitation tasks results, and quality of life assessment and supports those professionals to make informed decisions on rehabilitation planning and personalisation as well as remote assistance and support action triggering. The scalable and robust cloud storage of data and ubiquitous web access provides the needed flexibility in order to get the maximum potential out of the telemonitoring and home support features because the therapist can access the station at any moment with any device that is connected to the Internet.

### 3.2. The Provided Services

As shown in Figure 1, the system offers a set of flexible and extensible services: Smart Home control, cognitive stimulation, and web access. Smart Home control service is aimed at giving control over the environment, as well as over useful devices. Thanks to that service, the user is able to control home devices (e.g., a light, a fan, and a radio) as well as to interact with the XBMC multimedia player. Cognitive stimulation services allow users to improve their cognitive capabilities by performing cognitive rehabilitation tasks assigned by a therapist or by using their creative skills through Brain Painting. Web access services enable participation and inclusion by offering users the possibility to engage in social interaction through the web, such as web browsing, emailing, and twittering.

More details about the functioning of these services are provided below in Section 5.

## 4. Materials and Methods

UCD is a process of engagement with target end users that adopts a range of methods to place those who may benefit most from the technology at the centre of the design process in terms of development and evaluation. Thus, the system development has been iterative and incremental to enable reflections with users, their families, caregivers, and therapists prior to implementation of the final system [13]. UCD has been adopted at each phase of the system definition and implementation in order to take into account users' feedback to have a solution that reflects users' requirements, needs and preferences.

**Figure 2:**
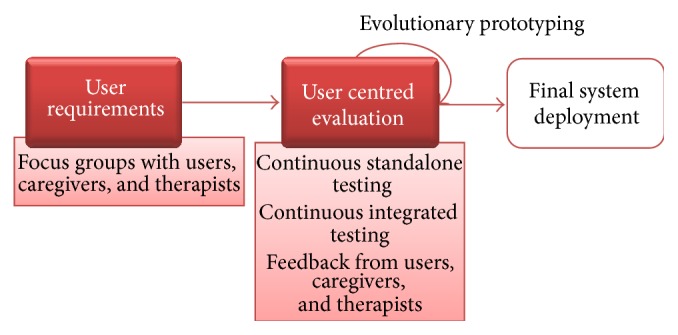
The adopted user centred design (UCD) approach.

Figure 2 sketches the adopted approach: first user requirements have been gathered, and then, users' evaluation is taken into account to improve the system, according to an evolutionary prototyping approach. Finally, the final system is deployed.

### 4.1. Ethical Issues

UCD is important to gather the preferences of target end users in the design and development of emerging technologies. The novelty within this approach is successfully gaining ethical approval to work directly with target end users, that is, people who have acquired brain injury. The challenges are many to engage in such innovative research with people who are considered to be more vulnerable than the general population. Achieving this helps align technical innovation to end user needs, increasing the likelihood of adoption and sustained use. The very fact that the technology is in the design and evolving phase brings a host of ethical issues to consider, which may be more crystallized when the target end users are considered to have cognitive impairment. An ethical framework developed within the project created a robust structure to unpin the research approach.

The core ethical principles of autonomy, beneficence, nonmaleficence, and justice were kept central to this framework. European legislation, international conventions, and local ethics committees were also incorporated.

The selection of appropriate end users was important within the ethical framework developed. People with neurological conditions including acquired brain injury (ABI) and amyotrophic lateral sclerosis (ALS) are considered vulnerable groups and it is imperative to safeguard their wellbeing. It is important that potential end users have a thorough understanding of the device and what it can actually do rather than what can be perceived as a result of film or the media. Additionally, informed consent is central to the ethical framework. Key workers within the end user organizations were contacted and asked to make the first contact with potential participants to identify those interested. Next an initial meeting was undertaken to introduce the potential participants to the project, watch videos of BCI, and ask questions before they were given an information leaflet to consider. A cooling off period of one week was given before the potential participant was contacted to assess her/his interest and desire to participate and give consent. Once the consent form was signed participants were invited to an interview to identify if the participant met the inclusion criteria. It is highly probable within BackHome as we are recruiting participants for home-based evaluation that not all participants will be able to give verbal or written consent. The researchers would incorporate a number measures to try to establish consent in this instant and may also have to consult with the family, and key caregivers when seeking consent from this target participant group.

Maintaining realistic expectations around the project is essential. Recent media attention around BCI technologies has portrayed the systems as a life changing assistive device [43]. However, BCI is not currently an off the shelf solution or a cure for a person with very limited mobility [44]. It is important to keep the perception of the systems functionally at an achievable level in line with the current constraints. Since even in a healthy population a small number of people are unable to operate a BCI, this technology is not going to be a potential solution for everyone and this can lead to feelings of frustration and disappointment. The system can be equally frustrating when it fails to respond to the user as accurately and quickly as they would like. Importantly, end users, family members, and caregivers must also be informed that participants will only have access to the BCI during their involvement in BackHome. This can become challenging if the technology provides a real improvement in a person communication and quality of life.

### 4.2. User Requirements

We collected the user requirements that emerged from focus groups with potential target end users, family members, caregivers, and therapists and from prior studies involving users of AT in general and BCIs in particular. We then created a prioritized list that contains the requirements in order of importance for the prototypes (see Table 1).

We assigned effectiveness to the highest priority, which goes hand in hand with functionality. The user should be able to achieve the desired task with satisfactory accuracy from the system and this is a prerequisite for its usability. Further, the system stability is of utmost importance for independent home use and has been critical during the UCD testing phases. Since the need of the user to request software support should be kept at a minimum, only those applications that have run stable during previous testing phases will be deployed and tested in the final independent home use testing. A key theme that emerged during the focus groups and evaluation phase and has become a crucial objective of the BackHome project is to improve the ease of use of the system. For this matter, both the end user interface that is used to control the applications and the caregiver interface used to launch the system need to be simple enough to be operated without requiring much training. Also the BCI hardware needs to be easy to set up. In order to assure that the main caregiver user interface will be easier to operate, mockups were evaluated by BCI experts and healthcare professionals. The goal was to have a “one-click interface” once the initial setup is done and the caregivers have been trained. Regarding the BCI hardware, during the focus groups phase users criticized the aesthetic appearance of the electrode cap as well as the limited mobility of the system. Users were, however, not involved in the aesthetic design of the cap, since good signal quality and thus effectiveness has a higher priority than the visual appearance. The dry electrodes should help to further reduce the time-needed for the setup of the system. For both the hardware and software setup the recommendation was to create an easy to understand user guide including a FAQ Section explaining the solutions to the most common problems and a video that explains the setup of the system visually.

Of course safety and privacy concerns that were raised during the discussions are of importance, but we did not list them on top of the priority list, because the necessary measures were already undertaken to assure both. Regarding the safety of the system, the EEG hardware needs to be certified as a medical device. Nevertheless precautions will be taken during testing with end users, for instance, no person suffering from epilepsy should use the system because of the fast flashing of rows and columns of the control matrix. To assure the privacy and data security of the study participants, we use a hypertext transfer protocol secure (HTTPS) for data that will be transmitted over the Internet. All experimental procedures need to be previously approved by the local ethics committees. To increase acceptance of the system, these measures need to be communicated to the study participants, their caregivers, and family members.

Independent use means that the BCI can be used without the need of an expert or the caregiver to be present, once the initial setup is complete as the end users will always need the help of another person (i.e., a caregiver or family member) to setup the BCI hardware and software. To promote the autonomy of the user, the navigation should be easy and enable the user to independently switch from one application to the next.

Last but not least, users asked for the possibility to personalize the system and adjust it to their specific needs. For instance, they suggested using shortcuts to frequently used applications and actions or the option to select prerecorded sentences.

### 4.3. User Centred Evaluation

Our UCD-based system stems from the engagement with target end users at all stages of the development and design of the system. The effectiveness of the system is identified by recording how accurate the system was at responded the user. The effort and workload required to operate the system is defined as efficiency and this was identified within an electronic version of the NASA-TLX. This test delivers a task load index which can be used for comparing the effort necessary to operate each tested system. Satisfaction with the system was measured by a visual analogue scale (VAS). This quick method has brought insight into general satisfaction with the system during continuous use. It informs us about the likelihood that the system will be used regularly. Additionally, a customized usability questionnaire was developed to identify user preferences with the system and an extended version of the Quebec User Evaluation of Satisfaction with Assistive Technology (QUEST 2.0 [29]) adapted for BCI research was administered. The rendered version (extended Quest 2.0 [20]) asks users to rate their satisfaction with different aspects of the BCI system, such as dimensions and weight of the system, comfort, ease of use, and effectiveness. This test evaluates 12 categories (namely dimension, weight, adjustability, safety, ease of use, well-comfort, effectiveness, service features, reliability/robustness, speed, learnability, and aesthetic of design) on a scale from one (“not satisfied”) to five (“very satisfied”). Participants were asked to complete the VAS after each interaction with the BCI and all other questionnaires were completed at the end of the evaluation cycle.

#### 4.3.1. New and Better Integrated Practical Electrodes

Several commercially available electrodes and amplifiers were tested to identify the most user friendly and reliable solutions for the final system. This testing assessed key issues like preparation time, signal quality, comfort, and robustness to noise but also technical aspects like signal to noise ratios, delay times, amplifier quality, and frequency response.

**Figure 3:**
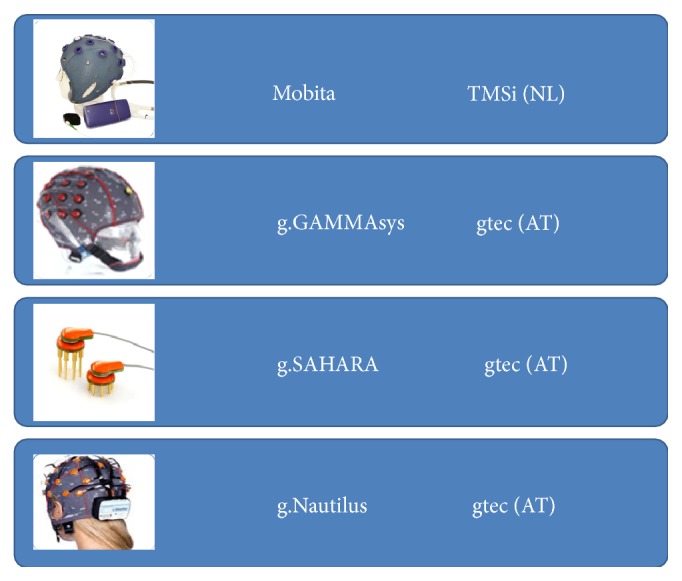
Overview of electrode systems.

After initial tests some insufficient electrode systems were excluded and the remaining systems (see Figure 3) underwent further advanced tests. The advanced testing phase included different tasks and environments in laboratory settings as well as testing with target end users. The main goal of this testing was to identify which practical electrode systems function best in different tasks and settings to find avenues for development and improvement. Figure 3 shows the final systems, namely, two dry, one wet, and one gel-based system, which were included in the advanced testing phase.

First, the preparation phase included the mounting of each EEG cap and the instruction of the participants. Second, the experimental protocol containing five parts is as follows.

*(1) Training*. The word “BRAIN” was used to train the classifier. The speller matrix consisted of six rows and six columns and every target letter was highlighted 30 times.

*(2) First Copy Spelling Run*. The participants had to spell the words “SONNE” and “BLUME.” Each single word was told to them shortly before they started spelling. If the user had selected a wrong letter s/he was advised not to correct it. The matrix for training and copy-spelling was the same.

*(3) Multimedia Player Run*. In this task the subject had to start a slideshow and to look at certain pictures within the XBMC multimedia player. Every command was announced by the supervisor. Wrong selections were corrected by announcing a correct alternative or the way back to the last correct selection. If the goal could not be reached within 15 selections the task was aborted. 

*(4) Internet Browser Run*. The goal of this task was to navigate to the Wikipedia article about BCI and at the whole article. Every command was announced by the supervisor. Ideally the task could be finished within ten to twelve correct selections. Wrong selections were corrected in the same manner as during the media player task. If the goal could not be reached within 18 selections the task was aborted. The matrix for this task had six rows and a variable number of columns depending on the amount of links on the actual webpage, with a maximum number of 7 columns.

*(5) Second Copy Spelling Run*. This task was performed in the same way as the first copy spelling task. The only difference was that two other words, namely, “TRAUM” and “KRAFT,” were spelled.

We tested the systems with seven preliminary test subjects and the performed tests provided useful feedback about features of interest, weaknesses, and problems of the different systems.

The evaluation of the VAS test reveals a high satisfaction of the users with all systems. Some users scaled systems with lower than 0.5 which means “not satisfied.” The three most important features, evaluated by the eQUEST2.0 questionnaire, are (1) speed, (2) effectiveness, and (3) durability together with learnability. The values for the most important feature, speed, were between 3.3 and 3.7. These values were lower compared to the values of the other eleven questions, so the participants were mainly unsatisfied with the speed. During the following advanced testing period the focus was on increasing the speed of the system. Regarding the electrode system, increasing the speed means reducing the time needed for the setup of the system and improves the signal quality.

#### 4.3.2. Friendlier and More Flexible BCI Software

The provided services have been tested separately before integrating them into the overall system. During the development phase, an evolutionary approach has been followed for the definition, implementation, and integration of the services. The corresponding feedback was passed on to the software developers and was carefully acknowledged for the implementation of the final system.

The evaluation of the provided services already integrated in the overall system was undertaken by a control group of preliminary test users and subsequently brought to target end users. Fourteen test users participated in the study as a control group (9 females, M = 28.1 years ± 8.6, range: 21–46) [45]. Five of the fourteen participants (4 female, M = 36.6 years, ±9.3, range 46–27) completed the test protocol on three different occasions, and the others performed the test protocol only once. A total of nine end users were recruited for the evaluation of the overall system. Four end users with muscle impairments (f, 80 years; f, 58 years; m, 42 years; m, 51 years) tested the BCI on one occasion each. Additionally, five end users (1 female, M = 37 years, ±8.7) who are living with acquired brain injury (Post ABI M = 9.8 years, ±3.7) completed the protocol on three different occasions. They tested the spelling application, used the web browser to post a Twitter message, performed the first level of the find-a-category and the memory cards task, and controlled a webcam. They switched between the different applications by selecting the corresponding symbols from the control matrices. The minimum number of selections that were necessary to complete the tasks ranged from 3 selections (webcam) to 18 selections (web browser/twitter). 

**Figure 4:**
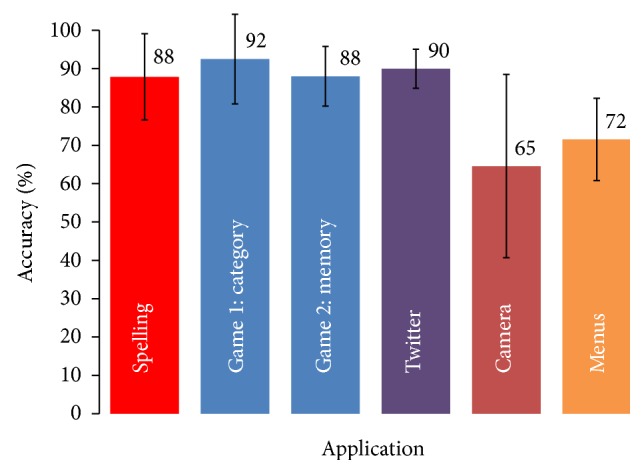
Average accuracies for the first session achieved by preliminary test users for the five tasks and for switching between tasks (menus).

*(1) Results with Preliminary Test Users*. The accuracies achieved by the fourteen preliminary test users during the initial BCI session for the individual tasks are depicted in Figure 4. Across all tasks average accuracies were >85% for the five test users that performed 3 sessions, and an average of 83% was achieved across the four applications. The accuracy scores across the three sessions remained relatively stable with the average variance <10%. The eQuest2.0 questionnaire revealed that users were quite satisfied with the system and the overall satisfaction score as rated with the VAS was also high (7.48). Through eQuest2.0 they indicated, however, that effectiveness, ease of use, and learnability were of the highest importance to them. A major issue was the reliability of the software that had to be restarted at times and a minor concern was the design of the control matrices that contained some “pixelated” symbols.

*(2) Results with Target End Users*. Seven of the nine target end users involved in the evaluation were able to gain control over the BCI and achieved satisfactory accuracies. Among the end users with muscle impairments, one achieved scores in advance of >90% accuracy for the spelling, games, and twitter tasks. Another achieved lower accuracies scores averaging 75% although overall she was pleased with the systems performance on the eQUEST2.0. Two of the initial four end users undertaking the evaluation on one occasion each were unable to operate the BCI. One end user had difficulty focusing on the relatively small symbols in the control matrices and the second had significant muscle spasticity that caused artifacts in the signal. The extended evaluation with end users with acquired brain injury recorded an average accuracy score of 76% across the four applications. The highest overall accuracy was achieved with the Speller (82.07% ± 13.34) and the lowest with the camera task (64% ± 22.8).

Overall, the end users indicated that they were more or less to quite satisfied on the eQuest2.0. Additionally, satisfaction with the BCI on the VAS was reported as high (7.64). The key recommendations from end users included the system being easier to use, reducing fatigue, and enabling independent use once the cap was placed and more effective selections. However, users also stated that stability of the software should be improved and that the time needed for one selection was still slow. The items that were rated as most important on the eQuest2.0 were speed, ease of use, effectiveness, reliability, and comfort. 

*(3) Evaluation with Caregivers and Family Members*. The final system is meant to be used in a home environment without direct supervision by the BCI researchers. Therefore, the BCI system must be incorporated into the daily routine of the caregivers and family members. The setup of the system should therefore be quick and the main software interface should be easy to configure and not require particular technical skills. Once configured, the system should ideally be a “one-click interface.” Hence, in the phase towards the evaluation during independent home use, the focus was more than before on the caregivers and family members, who need to be able to setup the BCI system on their own.

The findings of the evaluation outlined that seven key areas were important to enhance the user interface: simplification of the interface for the user, the terms and language used, the design, ability to navigate the interface, feedback from the interface, signal acquisition, and the ability to personalize the caregiver interface. Very clear and simple steps through the interface to help in the setup of the BCI were recommended. Recommendations such as a start-up checklist, troubleshooting support when an issue emerges and video instructions were outlined. The terms and language used were also important. Nontechnical terms must be used plus the ability to get an explanation by hovering the mouse over the term or click on the icon to find out more. Navigating the interface and the overall design were still not intuitive. Questions were also raised about the feedback the user would get from the system such as what happens when one electrode does not have satisfactory signal. And whether there is enough information for the user to be able to rectify a problem that occurs. Additionally, during testing signal acquisition and quality emerged as one of the most challenging issues particularly for nonexpert users. Signal acquisition is fundamental to BCI control and needs to be achieved in the most simplified form with additional information when the user is finding it more difficult. 

*(4) Evaluation with Therapists*. Following on from the initial gathering of user requirements of therapists, we have developed research collaboration with neurological occupational therapists and speech and language therapists. The therapists met with the researchers on four occasions (*N* = 10; *N* = 9; *N* = 3; *N* = 3) to help in the definition and development of the cognitive rehabilitation tasks. The technical developers integrated the outcome from the meetings into the application in an iterative process to be reviewed by therapists at the next session.

The therapists provided invaluable input into the cognitive rehabilitation in terms of the usefulness of the task, difficulty levels, presentation, language, outcomes, sequencing, number of steps, and application to practice. A framework for the development of cognitive skills has been created against which cognitive tasks were mapped during the collaborative stage of development. The domains emerging within the framework reflect various levels of cognitive complexity [46]: perception, attention and concentration, memory, and executive functions.

A total of 53 therapists took part in an overall evaluation of the services offered by the therapist station. Following a presentation of BackHome functionalities each participant was asked to evaluate the platform in terms of its application to everyday community based practice on a specifically developed usability questionnaire. Overwhelming, therapists reported that the station would facilitate their day-to-day practice and benefit their client. Recommendations included having a greater range of cognitive rehabilitation tasks increase the clarity of the results and instructions to support therapists without IT skills.

Subsequently, 36 of the therapists completed a protocol on the station which included setting up a client as a user, scheduling quality of life assessments and cognitive rehabilitation sessions, and reviewing the results of a clients scheduled assessment and their cognitive rehabilitation session. Each therapist then completed a second questionnaire to evaluate the technical components of using the therapist station. Therapists evaluated positively the therapist station, and they found it easy to use and appreciated both functionality and graphical aspect.

#### 4.3.3. Better Telemonitoring and Home Support

Also in the conception and development of the TMHSS a UCD methodology has been adopted. Similarly to [47], questionnaires and focus groups in relation to general requirements of the sensor-based TMHSS were run during the system design early stages. Participants were asked about its general requirements. For example, “How would you like to get monitored your physical and psychological status?”; or “What would you like to have at home to feel more secure, video cameras detecting intruders, sensors identifying anomalies?”. The same participants met four times in two hours sessions to discuss aspects related to the different technical issues and to assure consistent results. The main objective was to extract information from users related to the interaction modes and also about sensors and devices preferences in relation to the general requirements of the system. A moderator carried out the sessions and an observer took field notes.

General results from the questionnaire showed that users considered telemonitoring and home support as an opportunity to facilitate their daily life, they felt quite confident with the use of technology, and they preferred easy to use devices and adapted interfaces. Participants reported that data collected by the sensor-based system should be provided in a secure and simple way, being worried about their privacy and the use of their personal information.

According to the evolutionary prototyping, a first version of the implemented sensor-based system was installed, tested, and evaluated in a test user's home in Barcelona. The corresponding user is a 40-year-old woman who lives alone [48]. On the one hand, collected data has been used to recognize habits as well as to a preliminary study aimed at assessing quality of life. On the other hand, feedback from the user has been used to improve the system in terms of performance and usability.

### 4.4. Recommendations

The outcome from the user centred evaluation of the system as well as the requirements gathered at the earlier stages of the development life cycle gives as output a list of recommendations that have been carefully addressed during the system development. Those recommendations, ordered according to the priority list, together with the corresponding user requirements, are shown in Table 1.

## 5. Results

As stated above, the main innovation driver of the BackHome project is the use of UCD for the first time throughout all development stages of a multifunctional BCI which has resulted in the delivery and deployment of the final BackHome system. The overall architecture result of the UCD process was depicted in Figure 1 and we have already labeled it as key innovation (i) of the project. We are presenting the results of our UCD innovation driver following the structure of the BackHome system architecture.

### 5.1. The User Station

#### 5.1.1. Practical Electrodes: The Wireless EEG Recording System

**Figure 5:**
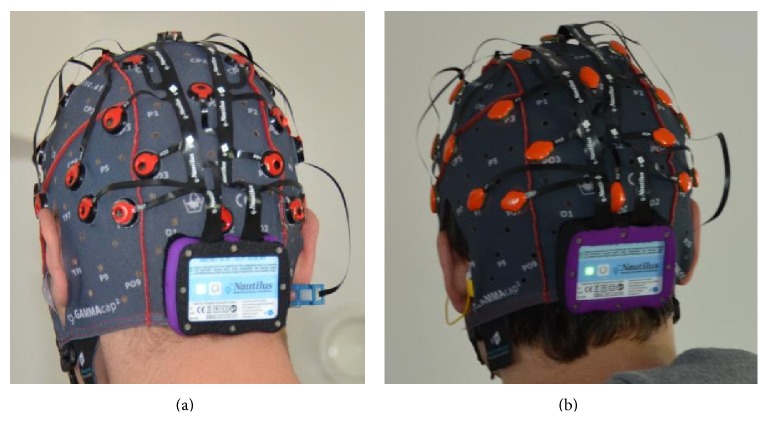
g.Nautilus headset with gel based (a) and dry electrodes (b).

Based upon the user feedback and the results of an advanced electrode testing described in Section 4.3.1, a new biosignal acquisition system called g.Nautilus was developed within the BackHome project. Its biosignal amplifier uses wireless technology to transmit the EEG signals with 24 bit resolution. The signal of each EEG channel is highly oversampled in order to keep the signal to noise ratio (SNR) high at the offered rates of 250 Hz and 500 Hz. Further it is capable of measuring the electrode-skin impedance at each electrode position for both gel based and dry electrodes (Figure 5).

A base station which is connected to the host system through USB is used to receive the recorded and digitized EEG signals. The biosignal amplification unit consists of the headset including the wireless biosignal amplifier electrodes, an EEG cap, and the base station including a USB cable for connecting it to the host computer and a QI compatible wireless charging station. The 34 electrodes including reference channel and ground are preconnected to the amplifier using a preconfigured set of electrode positions.

The user interface of the headset consists of the power switch and the status LED. Both are located on the top face of the head set. The electrodes are connected to two groups of monopolar amplifiers. The first group is connected to the ground electrode and the first 16 EEG channels and the second group is connected to the electrode positions 17 to 32 and the reference channel. The main advantage of this system which we labeled as key innovation (ii) is its wireless technology and the choice of dry electrodes. Both factors have often been criticized from test users as well as target end users when they used other nonwireless technologies. Most notably the fact that no hair wash is needed when using dry electrodes is a big increase in terms of usability. Furthermore the quick and easy montage of the system makes it also practical and comfortable especially for target end users.

#### 5.1.2. Easy to Use Software: The Implemented Services

This long section describes all the novelties related to the user friendly software to access multifunctional applications through BCI which we labeled as key innovation (iii). The BCI user interface is based upon the screen overlay control interface (SOCI) library [49], which allows embedding the BCI stimuli on top of the native interface of any user application and communicates with the BCI hardware via a network connection. Along with the integration of SOCI the primary user interface (PUI) has been redesigned according to the recommendations expressed by the users during the UCD tests summarized in Section 4.3.2. Now, for instance, the number of lines, columns, the spaces in between, and thus the display size of the individual icons are now defined depending upon users' capabilities. Masks which are smaller are automatically centred by SOCI within the visible area. Alternatively the new version of the Application ConTrol and Online Reconfiguration (ACTOR) protocol, described in [50], allows the application to explicitly position its masks on the screen.

**Figure 6:**
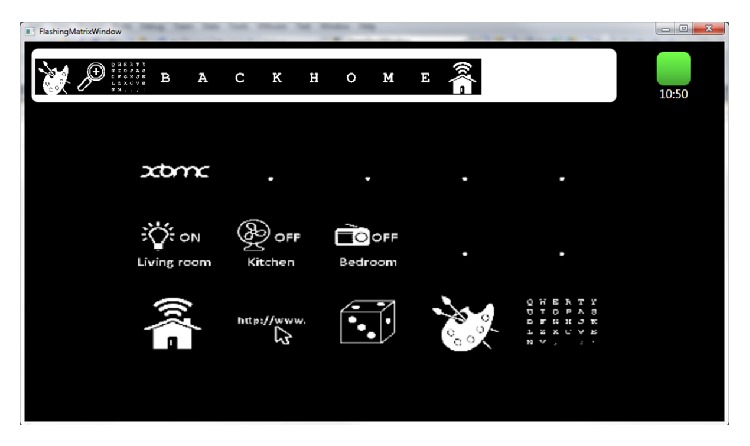
Primary user interface showing the main screen of the Smart Home service.

Figure 6 shows the PUI, which is split into three sections. On top of the screen a history bar shows the last selections made. On the top right side thereof the current time of the day is displayed along with the quality of the EEG signal recorded. The middle section displays the active P300 matrix for the currently selected service or service group, for example, the Smart Home group for interaction with user's environment and the multimedia player. The bottom row of the screen displays the menu which allows switching between the different services and service groups (i.e., Smart Home control, web access, Cognitive Rehabilitation Games, Brain Painting, and Speller). When the user selects one of the icons on the screen the system automatically activates the selected service, service group, and displays the corresponding masks. The masks available for each service and service group are described in detail along with each service provided by the BackHome system in the following sections.

**Figure 7:**
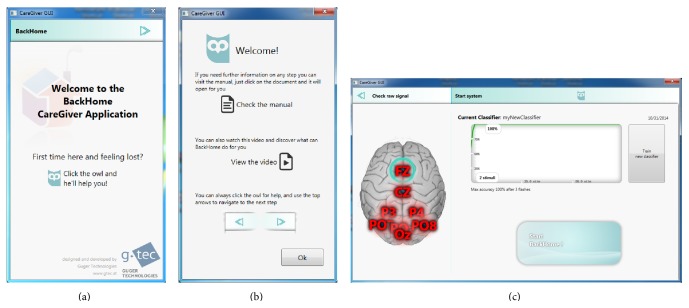
Care giver interface. The interface has been designed to optimally guide the care giver through the setup process step by step (a) and provide access to help and support information (b) on every screen. Only those information and controls are shown which are necessary to accomplish the current step (c) or advance to the next one when finished or go back to the previous one.

The user interface for the caregiver (see Figure 7) allows starting the system with just one-click, to create the classifier (in training mode) and to shutdown (Quit) the BCI system. Further when the system is started it automatically activates the check signals mode and starts the signal acquisition. This simplifies the mounting of the electrode cap as the signal quality display is visible during the whole procedure.

All the Smart Home control, cognitive stimulation, and web access services rely on a P300 spelling and control system. In the adopted speller the highlighting happens with freely selectable images (famous faces) instead of just changing the color of the background [51].

*(1) Smart Home Control*. Smart Home devices, which provide control over the software environment and free standing electrical goods, are installed in the user's home so that the user is able to control them through the BCI. The current system integrates ON/OFF switches and power meters connected with appliances (e.g., lights, radio players, etc.). In particular, we use Everspring AN158, a switch plug-in between standard wall outlets and appliances. It can switch ON/OFF as well as measure the electrical consumption. The end user controls the appliances via a P300 control interface (in the primary user interface in Figure 6, three appliances are shown: a light, a fan, and a radio, together with the information regarding their location in the house).

**Figure 8:**
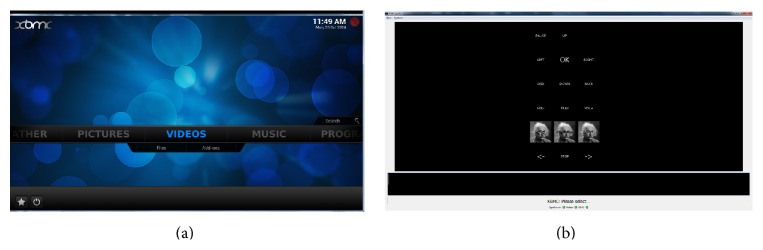
Screenshot of the XBMC application (a) and the P300 controller (b).

Apart from controlling devices through the P300 control interface, the user can also interact with a multimedia player to see photos, videos, movies, and so on. The implemented media player application is XBMC (available: http://xbmc.org/. [Accessed 20. 10. 2014]); see Figure 8. XBMC is a free and open source media player application and is designed to be controlled with a remote control or game controller. With these control devices the amount of input dimensions is very limited compared to mouse control or keyboard control, which is the main reason why we decided to use XBMC. Another feature which makes XBMC ideal to be controlled with our BCI system is the predefined network interface. We use a raw TCP socket based interface with a JavaScript Object Notation-Remote Procedure Call (JSON-RPC) protocol. A big advantage of this network based control is that XBMC can run on a different PC than the P300 system. This is due to the fact that the XBMC was designed for a game-console. 

*(2) Cognitive Stimulation*

**Figure 9:**
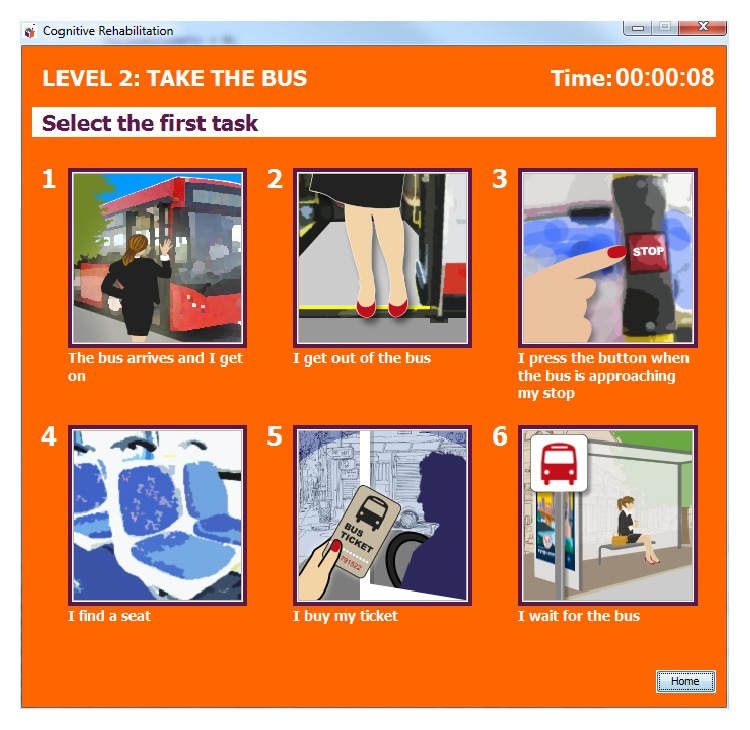
Daily-life activities game (level 2) screenshot.

*Cognitive Rehabilitation*. Together with occupational therapists and speech language therapists, three cognitive rehabilitation tasks have been defined, developed, and integrated defined to improve cognitive capabilities of users. Each game consists of a P300 matrix and an interface in which the game is shown (see Figure 9). Each task has three levels of difficulties that the therapist may select through the therapist station. Results are shown to the user after each play and are also sent back to the therapist who may control user's evolution. The implemented tasks are* memory cards*, aimed at enhancing memory skills [52];* find a category*, which provides users with activities for improving semantic and reasoning skills essential in cognitive rehabilitation, language, and learning; and* daily-life activities*, which allows users to develop skills that can be applied to real life tasks.

**Figure 10:**
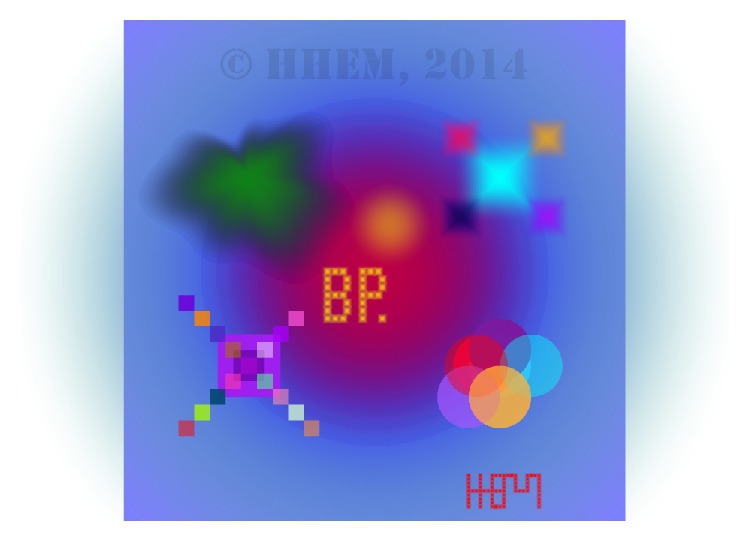
Brain Painting entitled “Die EU Muskeltiere, the EU Muscleteers,” with kind permission from the artist.

*Brain Painting*. The Brain Painting application allows users to create paintings on a virtual canvas. Selections are made via a 6 × 8 black and white, P300 based control matrix. It allows users to choose a grid size, move the cursor, choose object form, size, color, and level of transparency, and zoom in and out of the picture. Once the user chooses a specific color, the selected object appears on the screen. Erroneous selections can be corrected by selecting the undo button. The application was evaluated with preliminary test users [53] and target end users [54]. Within the project BackHome it has been installed at two target end users' homes. Results of this long term evaluation that is ongoing were reported in [55–57]. Both participants were diagnosed with amyotrophic lateral sclerosis (ALS) and have an artistic background. The first user (A) is a 74-year-old female and the second (B) is a 73-year-old male. After an initial phase in which users as well as caregivers and family members were trained to set up the system, they used it independently. In case of technical errors, they were solved via remote control. After every session users were prompted to indicate their perceived level of control, satisfaction, and frustration. User A painted for 403 hours in 271 sessions and user B painted for 65 hours in 58 sessions. Their overall satisfaction with the system during these sessions was high and in the majority of sessions, the level of control was rated as either medium or high by both users [57]. Since the installation in January 2012 user A has created numerous paintings that she presented at exhibitions in Easdale, Scotland, and Würzburg, Germany. One of her paintings was featured on the cover of the scientific journal Brain [58]. However, the impact the system has for these users is best expressed in a statement from her: “Brain Painting makes me happy and satisfied every time again, if it works-if not, then frustration and disappointment. Fortunately this happens in only few cases. Brain Painting completely changed my life. I can paint again and be creative again using colours […]” (Nov 2013, translation by Kübler et al., 2013 [58]).  Figure 10 shows a Brain Painting created by user A.

**Figure 11:**
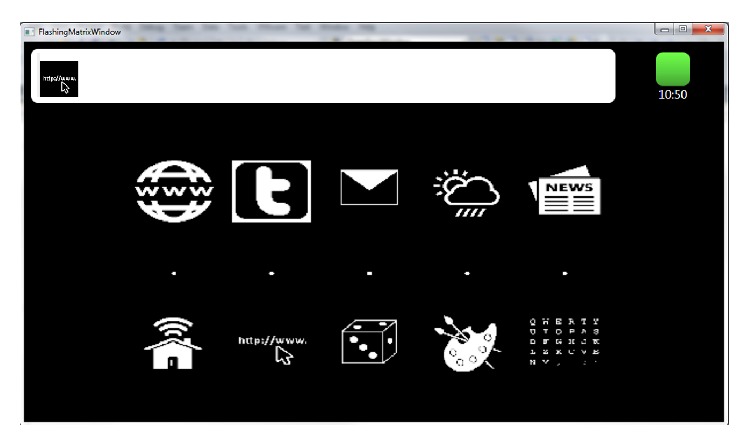
PUI of the web access services.

*(3) Web Access*. One of the main objectives of BackHome is to support people with limited mobility access the Internet and all the online services it has to offer to enhance social inclusion. Thus, services that allow users to access World Wide Web have been provided. In particular, a new web browser and an email reader have been implemented and integrated.  Figure 11 shows the primary user interface for web access services: web browser, Twitter, e-mail, and two personalized shortcuts, one linked to a weather forecasting service geolocalized depending on the installation and one linked to the preferred online newspaper.

*Web Browser*. The web browser was developed for the BackHome system using the Qt software framework. It enables bidirectional communication between the browser and the BCI system. Each hyperlink displayed on the webpage is assigned a letter (or a combination of letters for more than 26 links on a page) and depicted next to it. These hints are also displayed in the P300 control matrix. Thus, each control matrix contains only as many elements as links on the web page, plus an additional 15 control elements that were needed to navigate the webpage (e.g., scroll up/down), pausing the flashing to look at a page and other purposes. Pausing the flashing will be automated in the final version of the prototype by detecting whether the attention is directed at the matrix or the application. The control matrices can contain up to 84 (14 × 6) elements. Hence, a maximum of 69 hyperlinks can be selected per page. If a text entry field is selected, the letter and numeral matrix are automatically displayed. Compared to a similar P300 web browser proposed in [59], the selection time can be reduced by about 25% with the current implementation.

The web browser was evaluated both as a standalone version, together with the multimedia player and the P300 speller [60], and integrated into the current system. In the study by [60] preliminary test participants and target end users were asked to search for the word “BCI” with the web browser and navigate to the corresponding Wikipedia article. A minimum of 12 selections were needed to complete the task. In the study by [45], participants posted a short twitter message (a minimum 18 selections were needed). Preliminary test controls could operate the web browser with high accuracies (88% in [60] and 90% in [45]) and the majority of end users could also control it (with lower accuracies) and felt in control while using it.

**Figure 12:**
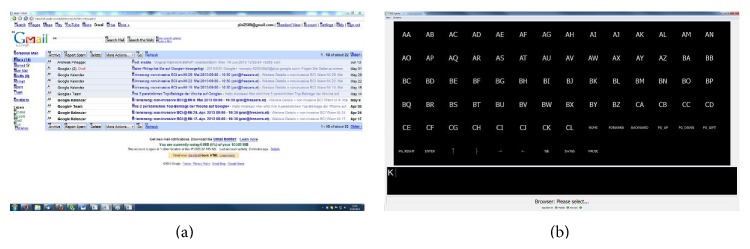
Screenshot of the Gmail home page (a) and the P300 controller (b).

*Email*. Email control was realized using the web browser described above. The challenge of this system is that webmail pages are very complex constructed and many of them use content dynamically created at the client-side. So far the link detector of the implemented web browser does not support such complex webpages. However, the system was successfully tested with the HTML version of Gmail (http://mail.google.com/); see Figure 12. Pinegger et al. showed in [61] that users can easily read and write emails with this system.

#### 5.1.3. The Telemonitoring and Home Support System

To monitor users at home, we develop a sensor-based system able to observe the evolution of the daily life activity of the user using a BCI [62], which we have labeled as key innovation (iv). The implemented system is able to monitor indoor activities by relying on a set of home automation sensors and outdoor activities by relying to MOVES (http://www.moves-app.com/).

Let us summarize here the main features of the implemented system, and the interested reader may refer to [48] for more details. As for indoor activities, we use presence sensors (i.e., Everspring SP103), to identify the room where the user is located (one sensor for each monitored room) as well as temperature, luminosity, and humidity of the corresponding room; a door sensor (i.e., Vision ZD 2012), to detect when the user enters or exits the premises; electrical power meters and switches, to control leisure activities (e.g., television and pc); pressure sensors (i.e., bed and seat sensors), to measure the time spent in bed and wheelchair. From a technological point of view, we use wireless z-wave sensors that send the retrieved data to a central unit located (based on Raspberry pi) at user's home. That central unit collects all the retrieved data and sends it to the cloud (service oriented WSO2 based infrastructure with APIs for third part integration) where it is processed, mined, and analyzed. Besides real sensors, the system also comprises “virtual devices,” software elements that mash together information from two or more sensors in order to make some inference and provide new information. In so doing, the sensor-based system is able to perform more actions and to be more adaptable to the context and the user's habits. Furthermore, the mesh of information coming from different sensors can provide useful information to the therapist. In other words, the aim of a virtual device is to provide useful information to track the activities and habits of the user, to send them back to the therapist through the therapist station, and to adapt the user station, with particular reference to its user interface, accordingly.

As for outdoor activities, we are currently using the user's smartphone as a sensor by relying to MOVES, an app for smartphones able to recognize physical activities (such as walking, running, and cycling) and movements by transportation. MOVES is also able to store information about the location in which the user is, as well as the corresponding performed route(s). MOVES provides an API through which it is possible to access all the collected data.

Information gathered by the sensor-based system is also used to provide context-awareness by relying on ambient intelligence [63]. In particular, thanks to the adopted sensors we provide adaptation, personalization, alarm triggering, and control over environment through a rule-based approach that relies on a suitable language [64]. Moreover, data collected by the sensor-based system has been used to automatically assess quality of life of people [65]; the interested reader may refer to [66] for a more deep explanation of the performed experiments.

### 5.2. The Therapist Station

The therapist station (https://station.backhome-fp7.eu:8443/BackHome/) is a web application that provides functionality for clinicians/therapists regarding user management, cognitive rehabilitation task management, and quality of life assessment, as well as communication between therapist and the end user using the BCI. Analogously to previous key innovations (i) to (iv), to our best knowledge, such therapist station connected to a BCI multifunctional system has not been implemented before, and we have labeled it as our key innovation (v). Therapists are able to interact with the users remotely in real time or asynchronously and monitor the use and outcomes of the cognitive rehabilitation tasks and quality of life assessment. It enables the therapist to plan, schedule, telemonitor, and personalize the prescription of cognitive rehabilitation tasks and quality of life questionnaires using the therapist station and facilitate the user to perform those tasks inside her/his therapeutic range (i.e., motivating and supporting her progress), in order to help to attain beneficial therapeutic results.

**Figure 13:**
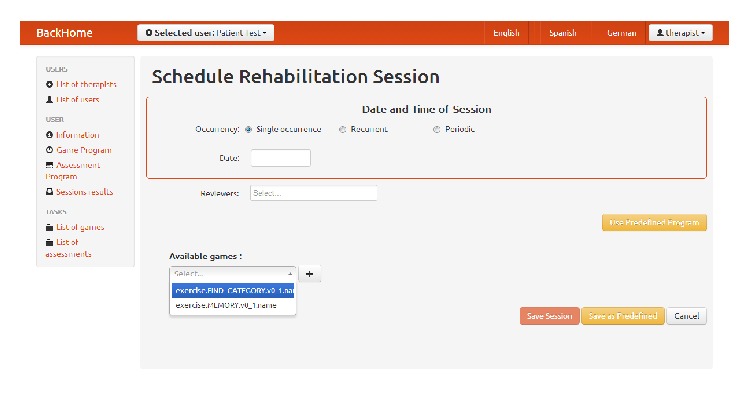
Scheduling cognitive rehabilitation tasks.

As for the cognitive rehabilitation sessions, using the therapist station, healthcare professionals can remotely manage a caseload of people recently discharged from acute sector care. They can prescribe and review rehabilitation sessions (see Figure 13). Through the therapist station, game sessions can be configured, setting the type of games that the user will execute, their order in the session and the difficulty level, and specific parameters for each one of them. Additionally, the therapist station allows healthcare professionals to establish an occurrence pattern for the session along the time. If the same session must be executed several times, professionals can set the type of occurrence and its pattern to make the session occurs at programmed times in the future. Once the session is scheduled, users will see their BCI matrix updated on the user station the day the session is scheduled. Through that icon, the user will start the session. The user can then execute all the games contained in it in consecutive order. Upon completion of the game session execution on user station, results are sent back to the therapist station for review. At this point, those healthcare professionals involved in the session—the prescriber and the specified reviewers—will be notified with an alert in the therapist station dashboard indicating that the user has completed the session. Healthcare professionals with the right credentials can browse user session results once they are received. The therapist station provides a session results view and an overview of completed sessions to map progress, which shows session parameters and statistics along the specific results.

One of the goals of the sensor-based system is to automatically assess quality of life of the users. Accordingly, results and statistics are sent to the therapist station in order to inform the therapist about improvement/worsening of user's quality of life (please refer to [65] for more details). Moreover, the therapist may directly ask the user to fill a questionnaire. The therapist can decide the occurrence of quality of life questionnaire filling and, once scheduled, the user receives an update in the BCI matrix. Once the user, with the help of the caregiver, has filled the questionnaire, results are sent to the therapist that may revise them.

Finally, through the therapist station, therapists may consult a summary of activities performed at home by the user; for example, visited rooms, sleeping hours, and time elapsed at home. Moreover, also the BCI usage is monitored and high-level statistics are provided. This information includes BCI session duration, setup time, and training time as well as the number of selections, the average elapsed time per selection, and a breakdown of the status of the session selections. Therapists have also the ability to browse the full list of selections executed by a user, such as context information as application running, selected value, grid size, and selected position.

## 6. Conclusions and Future Directions

The BackHome project was designed according to a user centred design approach, used for the first time throughout all development stages of a multifunctional BCI. Driven by that approach, the project achieved five key innovations: (i) an architecture able to meet the requirements of BCI multifunctionality and remote home support; (ii) a light, autonomous, comfortable, and reliable BCI equipment; (iii) an easy to use software to control multifunctional applications; (iv) a telemonitoring and home support system for BCI independent use; and (v) a therapist station to manage BCI-based remote services.

Currently, the system has been installed and is running in four target end users' homes in Belfast and one in Würzburg for a final period of testing. The main purpose of the on-going final testing is to assess user acceptance of the BackHome system when deployed in a continuous way in real world scenarios, that is, at users' homes, and to analyze the impact of the use of such technology on the quality of life of individuals in need. The potential socioeconomic impact of the exploitation of such a system as well as barriers and facilitators for future deployment and spreading of such technologies will therefore be analyzed and reported. In that sense the BackHome project is also adapting and testing the commercial telehealth product called HomePod aimed to remotely monitor and assist one target population of the BackHome project, stroke patients. This so-called StrokePod, which integrates sensors and services of the BackHome system, is a promising by-product of the project targeted to larger audiences.

We decided to discard the integration of other research advances explored in the project such as a multimodal fatigue support component and hybrid BCI capabilities because we considered that they were not mature enough for real world deployment, but they will be further researched and might be eventually integrated in the future.

This BackHome platform and the services provided make a significant contribution to enhancing the opportunities for social inclusion and eHealth for people living with physical and cognitive impairment who would otherwise struggle to exert autonomy and independence in a world that is much more digitally enabled than ever before.

## Figures and Tables

**Table 1:** Recommendations from users in order of priority.

Key aspects of the system	User requirements	Recommendations/planned system specs
Effectiveness	With the system users should achieve the task as accurately and completely as possible	Implementation of famous faces paradigm for the P300 matrix, dynamic stopping method, and error suppression

Reliability	The system must be stable during everyday use	Only those services that are stable (after an initial test with preliminary test users) will be evaluated during independent home Use/software bugs will be fixed during initial testing phase
Robustness	The system has to be robust with respect to anomalies, malfunctioning or in case some sensors or services stop working; in all those situations, the system should continue to work with reduced functionalities	

Functionality	The system should allow the user to perform as many (simple) tasks as possible to increase autonomy of user	Spelling, web browser, multimedia player, Cognitive Rehabilitation Games, Smart Home control, Brain Painting

Ease of use	(i) The system should be simple to operate (menus should be intuitive) (ii) The software and hardware must be easy to set-up by nonexperts (iii) Instructions on how to operate the system must be clear (iv) Minimal technical support should be necessary (technical problems should be resolvable via remote access)	(i) Assured through the evaluation of a mockup of the software (ii) A one click interface is planned (iii) An easy to understand user guide will be created/including video explanations

Efficiency	Workload and speed of communication should be at an acceptable level, ideally comparable or better than the AT in use	A dynamic stopping method during usage of the services will assure that only the necessary amount of repetitions will be used to maximize speed

Safety	The system must not constitute a risk to the health of the user; this concerns especially the safety of the individual parts (electrodes, wires, etc.)	The system does not constitute a risk to the health of the user; the hardware is CE certified/this will be communicated to increase its acceptance

Comfort	(i) The EEG cap should be comfortable to wear for several hours (ii) To prevent fatigue of one modality (e.g., eyestrain) users should be able to switch between different control signals (modes of control) (iii) Ideally no gel is necessary to acquire good EEG signals	(i) Only P300 will be implemented because this control signal offers the best compromise of accuracy and stability (ii) The developed EEG systems can be dry or gel-based depending on the users preferences

Privacy	(i) No information should be transmitted or be visible to persons except to those it is intended for or the user has agreed to share the information with (ii) Information that is transmitted wirelessly or via an internet connection must be transmitted and stored sufficiently securely	(i) A secure protocol will be used for transmission of data over the internet (hypertext transfer protocol secure (HTTPS)) (ii) No sensitive data will be transmitted wireless (iii) All information will be stored according to data protection laws

Mobility	(i) To allow for maximum mobility, the system should be small and wireless (ii) It should be possible to use the system in a lying position and/or if the user is seated (e.g., if the user is lying in his bed or sitting in a wheelchair)	The EEG system will be both small and wireless

Possibility of independent use	The user should be able to operate the system with as little help from the caregiver as possible	After the EEG setup is done and the system started, the user can switch between applications or pause the system by himself and because of the planned noncontrol state detection system, the BCI will refrain from making selections if the user is not concentrating on the control matrix

Aesthetic design	(i) The design of the interface should be appealing (ii) The design of the cap should be appealing and as inconspicuous as possible	(i) The design of the cap could not be influenced without compromising its functionality (ii) Size of the amplifier must be reduced and transmission to the computer is wireless

Configurable to the needs of the user	The system should contain the possibility to adjust the system to the needs of the individual user (e.g., store prerecorded sentences or implement shortcuts)	The system will cover many services already but the software will also be implemented such that it allows for extensibility with other applications that are desired by the user
